# Characterization of Physicians That Might Be Reluctant to Propose HIV Cure-Related Clinical Trials with Treatment Interruption to Their Patients? The ANRS-APSEC Study

**DOI:** 10.3390/vaccines8020334

**Published:** 2020-06-23

**Authors:** Christel Protiere, Lisa Fressard, Marion Mora, Laurence Meyer, Marie Préau, Marie Suzan-Monti, Jean-Daniel Lelièvre, Olivier Lambotte, Bruno Spire, the APSEC Study Group

**Affiliations:** 1 Aix Marseille University, INSERM, IRD, SESSTIM, Sciences Economiques & Sociales de la Santé & Traitement de l’Information Médicale, 13005 Marseille, France; lisa.fressard@inserm.fr (L.F.); marion.mora@inserm.fr (M.M.); marie.suzan@inserm.fr (M.S.-M.); bruno.spire@inserm.fr (B.S.); 2 INSERM, U1018, Université Paris-Sud 11, AP-HP, Hôpital de Bicêtre, Département D’épidémiologie, 94270 Le Kremlin-Bicêtre, France; laurence.meyer@inserm.fr; 3 GRePS, Lyon 2 Université, 69676 Bron, France; marie.preau@univ-lyon2.fr; 4 INSERM, U955, Equipe 16, Université Paris Est, Faculté de médecine, Vaccine Research Institute, 94000 Créteil, France; jean-daniel.lelievre@aphp.fr; 5 Assistance Publique-Hôpitaux de Paris, Hôpital Bicêtre, Service de Médecine Interne et Immunologie Clinique, INSERM, U1184, 94270 Le Kremlin-Bicêtre, France; olivier.lambotte@aphp.fr; 6 Immunology of Viral Infections and Autoimmune Diseases, Université Paris Sud, UMR 1184, 94270 Le Kremlin-Bicêtre, France; 7 CEA, DSV/iMETI, IDMIT, 92260 Fontenay-aux-Roses, France

**Keywords:** HIV cure research, HIV cure-related clinical trials, physicians, viewpoints, analytical treatment interruption studies, principal component analysis, multivariable linear regression

## Abstract

HIV cure-related clinical trials (HCRCT) with analytical antiretroviral treatment interruptions (ATIs) have become unavoidable. However, the limited benefits for participants and the risk of HIV transmission during ATI might negatively impact physicians’ motivations to propose HCRCT to patients. Between October 2016 and March 2017, 164 French HIV physicians were asked about their level of agreement with four viewpoints regarding HCRCT. A reluctance score was derived from their answers and factors associated with reluctance identified. Results showed the highest reluctance to propose HCRCT was among physicians with a less research-orientated professional activity, those not informing themselves about cure trials through scientific literature, and those who participated in trials because their department head asked them. Physicians’ perceptions of the impact of HIV on their patients’ lives were also associated with their motivation to propose HCRCT: those who considered that living with HIV means living with a secret were more motivated, while those worrying about the negative impact on person living with HIV’s professional lives were more reluctant. Our study highlighted the need to design a HCRCT that minimizes constraints for participants and for continuous training programs to help physicians keep up-to-date with recent advances in HIV cure research.

## 1. Introduction

Achieving a HIV cure has become a research priority, implying the need for HIV cure-related clinical trials (HCRCT) with analytical antiretroviral treatment interruption (ATI) [1,2,3,4,5,6,7,8,9,10,11,12,13]. Even though HIV is now a manageable chronic illness, people living with HIV (PLWH) have to take daily treatments for the rest of their lives. In addition to the difficulty of treatment adherence over such a long period, daily treatment is a constant reminder to the patient of the disease; it impacts quality of life and is associated with long-term comorbidities. Moreover, stigmatization [14,15] is still a concern, and combined antiretroviral therapy (cART) has serious economic consequences on public health [16].

However, in the current context of modern, well-tolerated cART, where new treatment regimens reducing administration frequency are being introduced, clinical and biological-related issues to HCRCT cannot be disconnected from associated ethical questions or, indeed, from the consequences on the daily lives of PLWH who will participate in HCRCT [17,18,19,20,21,22,23,24,25,26,27,28]. The limited direct benefits for participants and the risk of HIV transmission during ATI might negatively impact physicians’ motivations to propose participation in HCRCT with ATI to their patients [21]. In this context, the perspectives of PLWH, physicians, and clinical researchers should be taken into account both when designing HCRCT and during the recruitment process. Indeed, the decision on participation in HCRCT will not be made by PLWH alone but in collaboration with their HIV physicians. It is therefore important that the latter be as convinced as the former about HCRCT. Accordingly, gathering information about their expectations of HCRCT is crucial. Taking into account physicians’ viewpoints is all the more important considering the impact of the patient-physician relationship, which is reinforced by the chronic nature of HIV infection [19,22].

Very few studies have explored physicians’ perceptions regarding HCRCT and their willingness to propose participation to their patients. In those that have, it was not always possible to disentangle physicians’ perspectives from those of PLWH. When possible, results showed that PLWH and physicians did not have the same preferences regarding several HCRCT strategies [27]. Furthermore, qualitative and mixed-method studies have shown that some physicians were reluctant about HCRCT [19,29,30,31,32]. Surprisingly, in quantitative studies, the majority of physicians (from 59% to 97.5%) declared they would propose HCRCT to their patients [27,30]. This counter-intuitive disparity in results has also been found among PLWH, who expressed more reluctance in qualitative surveys [19,29,30,32,33,34] than in quantitative surveys [18,23,27,30,35,36]. Besides discrepancies between declared intentions of interest in participation and actual behavior [37], other possible explanations for this disparity were differences in methodology and in the questions asked regarding willingness to propose HCRCT [27]. 

To explore this disparity in greater detail, in a previous study [36], we compared the proportion of PLWH who declared their intention to participate in HCRCT using the usual direct question found in the literature: “If an HCRCT were available, would you participate in it?”, with the proportions inferred from a reluctance to participate score derived from their level of agreement with four viewpoints regarding HCRCT (see details in the Materials and Methods section) [30]. We found that results differed for each approach, suggesting an overestimation in quantitative studies of PLWH’s self-declared willingness to participate in HCRCT [18,23,30,35]. Since we hypothesized that results obtained with our novel approach may better reflect real-world decision-making, and may partly overcome the issue of social desirability bias [38,39], in the present study, we used the same methodology to explore physicians’ willingness to propose HCRCT with ATI to their patients.

This study had two objectives: (i) to define the proportion of HIV physicians who might be reluctant to propose participation in HCRCT with ATI to their patients and (ii) to acquire a greater understanding of the characteristics of physicians that would be more or less likely to propose participation.

## 2. Materials and Methods

ANRS-APSEC (Acceptability, expectations, and preferences for HCRCT among PLWH with undetectable viral load and caregivers) is a comprehensive cross-sectional French study comprising three steps (Figure S1). We present here analyses from one section of the third step, which examined HIV physicians’ level of agreement with four viewpoints about HCRCT.

The study received ethical approval from the CCTIRS (Advisory Committee on Information Processing of Research Information in the Field of Health) and the CNIL (National Commission for Computing and Liberties).

### 2.1. Survey Population and Data Collection

Between October 2016 and March 2017, in HIV departments from 24 centers across France, all HIV physicians present during a dedicated week were invited to participate in a survey that comprised a face-to-face computer-assisted questionnaire administered by trained interviewers. All the physicians received an information letter and provided written consent. 

### 2.2. Outcome

One questionnaire section presented physicians with four viewpoints (developed and drafted based on results collected during the second step of the ANRS-APSEC survey [30]), covering a spectrum from strong motivation to strong reluctance with regard to proposing participation in HCRCT with ATI to patients (described in Appendix A). Physicians were asked to rate, on a 7-point Likert scale, to what degree each of the four viewpoints reflected their opinion (coded from 1 = strongly disagree to 7 = strongly agree).

**Figure A1:**
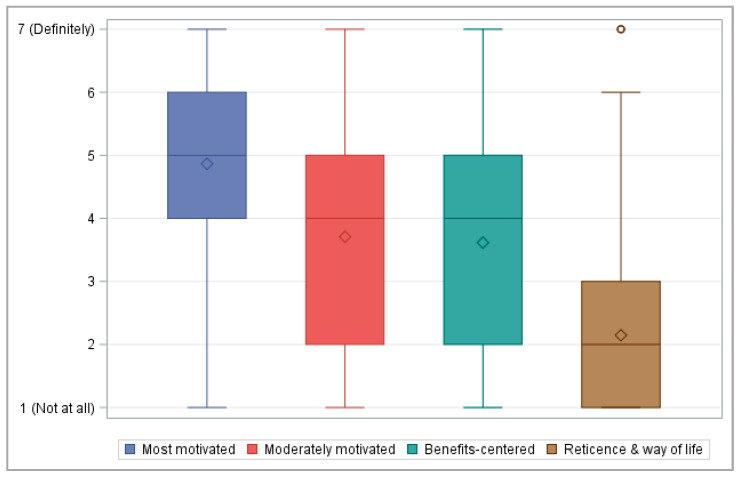
Respondents’ levels of agreement with the four viewpoints regarding HCRCT (ANRS-APSEC study, *n* = 164).

To construct the dependent variable (see Appendix B for details), we performed a principal component analysis of their answers, creating a “reluctance score” to represent their level of motivation to propose participation in HCRCT to their patients (from −1.7 very motivated to +3.3 very reluctant, Figure A1). The higher the score, the more reluctant physicians were to propose HCRCT. 

### 2.3. Explanatory Variables

Identity and sociodemographic variables included gender, age (continuous), being in a relationship (yes/no), feeling part of the LGBT (yes/no) or heterosexual (yes/no) communities, and self-identification as an HIV activist (yes, definitely/yes, somewhat/no).

Professional characteristics included specialty, number of years since graduating (continuous), academic or research involvement (yes/no), a sliding indictor for orientation of professional activity (from 0 = care to 10 = research), participation in prevention/information actions organized by HIV associations (yes/no), and requested to collaborate in the writing of France’s National HIV guidelines (yes/no).

The use of seven potential sources of information about HIV cure research was explored: international conferences, national conferences, meetings of learned societies, international scientific literature, HIV associations’ journals, internet websites, and participation in multidisciplinary networks (yes/no to each). A score measuring the number of sources of information was constructed by summing answers to these seven items.

Experience with clinical trials was explored though several items. The number of clinical trials physicians had participated in (continuous), general attitude about participation (favorable/rather reluctant/depended on the trial’s characteristics/deferred to the department head’s decision), and level of agreement with the statement that participation in clinical trials helps advance the work of previous generations (definitely agree/somewhat agree/somewhat disagree). With regards to the most recent clinical trial physicians had participated in, three reasons for participation were explored as follows: to help advance research, their department head asked them, out of interest (yes/no to each). Furthermore, physicians were asked whether they considered that they had adequately informed their patients of the most recent trial’s potential benefits and risks (definitely/somewhat/not enough). Physicians’ beliefs in the future availability of a HIV cure treatment within their career span were also explored (yes/no/do not know).

Physicians were asked about their perceptions of their patients’ levels of concern regarding their disease using fourteen HIV-related difficulties (not at all/a little/very concerned) as follows: shorter expected life, side effects related to current cART, severe fatigue, uncertain future, risk of HIV transmission, discrimination, difficulties constructing a stable relationship with a partner, having to live with a secret, feeling unable to lead a normal life, negative impact on health in general, negative impact on sexuality, negative impact on professional life, and cost for society and out-of-pocket expenses. Their perceptions of whether they considered that their patients cope with having to take cART on a daily basis (yes/no), and how uncomfortable their patients were with cART-related side effects (very/little/not at all), were also investigated.

Finally, the questionnaire included two items regarding current cART: physicians were asked whether they were very confident in them (yes/no) and whether they thought they would continue to be effective over the long term (yes/no).

### 2.4. Statistical Analyses

The reluctance score allowed us to infer the proportions of respondents that would be motivated or reluctant to propose HCRCT and to compare these proportions with those obtained when we used the usual direct question found in the literature “If an HCRCT were available, would you participate in it?” (“Absolutely not”, “Probably not”, “Yes, perhaps”, or “Yes, definitely”). This comparison was made by calculating, for each category of response to the usual direct question, the proportions of physicians with (i) a positive score (i.e., reluctant to participate), (ii) a score higher than the third quartile of responses (25% of the most reluctant), and (iii) a score higher than the 90th percentile of responses (10% of the most reluctant).

We performed a multivariable linear regression to determine factors associated with physicians’ reluctance to participate in HCRCT. Variables with a *p*-value < 0.20 in the univariable analyses were considered eligible for multivariable testing. A stepwise backward selection procedure with a threshold at *p* = 0.05 was used to define the ones staying in the final multivariable model. All analyses were based on two-sided *p*-values, with *p* < 0.05 indicating statistical significance. They were performed using SAS 9.4 statistical software (SAS Institute, Cary, NC, USA).

## 3. Results

All physicians enrolled in the ANRS-APSEC study were included in the present analysis (*n* = 164), with no missing data regarding the level of agreement with each of the four viewpoints about HCRCT with ATI to their patients. Participants’ median age was 50 (interquartile range (IQR) 41–57) years, 51% were women, and 9% reported feeling they belonged to the LGBT community, while 79% felt they were part of the heterosexual community (Table S1). Most of the study sample were infectiologists/immunologists (51%). The median number of years since graduating was 21 (IQR 10–28). Their activity was mostly care-orientated (median of 3/10 (IQR 2–5)). The main sources of information about HIV cure research reported by physicians included international scientific literature (87%) and national conferences (85%), while HIV associations’ journals were less frequent (43%). Overall, physicians reported a median of five (IQR four to six) information sources out of the seven proposed.

Physicians reported having participated in a median of 20 (IQR 10–40) clinical trials. They mostly (65%) agreed that participating in clinical trials helps advance the work of previous generations. However, with regards to their general attitude regarding participation in clinical trials, few indicated they were favorable (14%), the majority indicating that participation depended on the trials’ characteristics (65%). Almost one-third reported that they had participated in their most recent clinical trial because their department head asked them.

### 3.1. Comparison between the Reluctance Score and the Usual Direct Question

**Figure 1:**
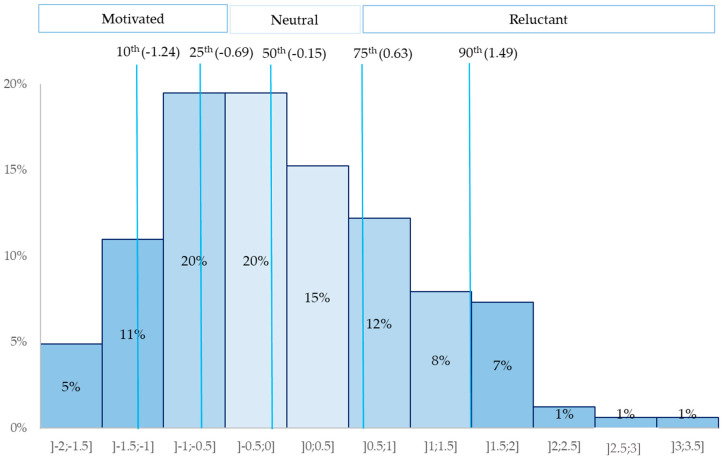
Distribution of respondents according to the reluctance score (*n* = 164).

The intention to propose participation in HCRCT with ATI using the direct question was distributed as follows: 61.25% responded “Yes, definitely”, 36.25% “Yes, perhaps”, 2.50% “Probably not”, and none “Absolutely not”. More nuanced replies were obtained when inferred from the reluctance score (RS). Indeed, based on a graphical reading of Figure 1 thanks to the cut lines representing percentiles, one can see that at least 10% of the physicians were part of the 10% most reluctant (RS ≥ 90th percentile) about HCRCT and that, overall, 18% to 30% were reluctant (RS around the 75th). Similarly, one can see that less than 16% were very motivated (RS around the 10th) and that overall less than 36% were motivated (RS around the 25th) about HCRCT. Finally, at least 35% of the physicians’ sample can be considered as neutral or hesitant. 

When we compare results from answers to the direct question with results from the reluctance score (Table 1), we can see a concordant trend: physicians who declared they would definitely propose HCRCT had a negative mean score, while the less sure a physician was, the higher the mean score. Among the 58 participants who declared that they would perhaps propose HCRCT, 64% had a positive reluctance score. Of these, 43% were part of the 25% most-reluctant respondents, and almost one in five were part of the 10% most-reluctant respondents. Their mean score was positive (0.4 ± 1.0). To a lesser extent, similar results were observed among the 98 physicians who declared that they would definitely propose HCRCT: 31% had a positive reluctance score, with 12% being part of the 25% most-reluctant respondents, and 3% being part of the 10% most-reluctant respondents. One can observe that none of the four respondents who declared they would probably not propose HCRCT were part of the motivated.

### 3.2. Factors Associated with the Reluctance Score

Table 2 presents the estimation of the final model with the variables retained according to the stepwise backward selection procedure (univariable analyses are presented in Table S2).

The final multivariable model showed that physicians who reported participating in the most recent clinical trial because their department head asked them, and those who believed that their patients were concerned (a little or very) about the negative impact of HIV on their professional lives would be more reluctant to propose HCRCT with ATI to their patients.

In contrast, physicians in general were favorable about participating in clinical trials; those who reported that their activity was more research-orientated, those who participated in actions organized by HIV associations, and those who informed themselves about HIV cure research with international scientific literature and HIV associations’ journals would all be less reluctant—that is to say, more motivated—to propose HCRCT to their patients. Finally, physicians who believed that their patients were very concerned about having to live with a secret would be less reluctant to propose participation in HCRCT.

Identity and sociodemographic characteristics were not associated with the reluctance to propose HCRCT. Neither was confidence in current cART or the belief that an HIV cure treatment would become available during their career span.

## 4. Discussion

From a public health perspective, the need to achieve a scalable HIV cure is widely acknowledged in order to reduce the chain of transmission, given that effective cART is not yet available for all PLWH and that the reduction in HIV incidence remains insufficient in certain key populations [8,40,41,42]. From an individual perspective, the lack of a cure implies life-long daily treatments with the threat of ART-related toxicities. As part of the wider research aimed at discovering how best to implement future HCRCT, the present study provides new insights to help better understand the characteristics of HIV physicians who might be reluctant or motivated to propose HCRCT to their patients and in which proportions. This study builds on existing literature—first, because it surveyed French HIV physicians from different departments caring for PLWH, including physicians not involved in HIV cure research, and second, because it provided a novel approach to infer physicians’ willingness to propose HCRCT.

Our results showed that physicians generally favorable to clinical trials were also favorable to HCRCT. However, they also showed the importance of involving all physicians—in particular, those whose professional activity is less research-orientated and those who do not inform themselves about HIV cure trials through scientific literature (perhaps because they might have less available time to inform themselves, or perhaps because of a lack of interest). Indeed, to ensure successful recruitment in HCRCT, the active participation of both physicians and PLWH will be required. Physicians need to be convinced for themselves of the possible benefits HCRCT will bring, and hierarchy-based decision-making cannot be sufficient. This is especially true given the ever-growing movement towards joint mobilization in HIV research [17,21,30,43,44] and highlights the need for continuous training programs, ensuring physicians are helped to keep up-to-date with recent advances in HIV cure research.

Moreover, our results also showed that the perceptions physicians had of the impact of HIV seropositivity on their patients’ lives were associated with their motivation to propose HCRCT. This result is particularly relevant in the context of HCRCT where several hospital visits and examinations are required—in particular, during the ATI period. Accordingly, physicians who considered that living with HIV is difficult because it means living with a secret would be more motivated to propose HCRCT in the hopes that it would release PLWH of this burden [30], while those worried about the negative impact on PLWH’s professional lives would be more reluctant. This result highlights the need to carefully design HCRCT that are as least-constraining as possible while guaranteeing patient safety and to find innovative solutions, such as tests at home to measure the viral load. These results can be linked to those observed for PLWH in other studies, including previous studies from ANRS-APSEC. First, HCRCT-related burdens—in particular, in terms of consultation frequency and, more generally, in terms of the deterioration of their quality of life [30,31]—have been found to be more decisive criterions than HCRCT outcomes in PLWH’s decisions to participate or not in HCRCT [27,29]. Second, financially precarious PLWH have been shown to be more reluctant to participate in HCRCT, perhaps because they may anticipate difficulties regarding hospital visits in terms of time and economic burden [36].

Finally, our results confirmed those obtained from PLWH regarding the possible overestimation of the proportion of respondents willing to participate in HCRCT when using the usual direct question [36]. Similarly, results were more nuanced when using the reluctance score derived from the level of agreement with four viewpoints regarding HCRCT [30]. Indeed, they suggested that, in reality, a greater proportion of physicians would probably refuse to propose HCRCT. This would seem to confirm our previous hypothesis (i.e., results obtained with our reluctance score methodology to estimating the willingness to participate may better reflect real-world decision-making and may partly overcome the issue of social desirability bias [38,39]). Indeed, some studies have already shown that measurement methods that are more cognitively involving than simply asking one direct question foster a greater understanding of respondents’ perceptions [45,46].

Besides the usual restrictions related to declarative data, the present study has other limitations. First, using a Likert scale might have introduced a central tendency bias [47]. However, the distribution of answers (Appendix B) suggests a lack of evidence of such a bias. Second, our results are linked to the French health system, where PrEP is universally available free of charge [48,49]. This fact might have been a positive signal that influenced our study sample’s responses, given that one of the main negative consequences related to HCRCT is an increased risk of HIV transmission during ATI [3,4,50]. Consequently, our results might not be generalizable to the whole PLHW population in France. Additional research is needed—not only in France but in other countries, especially were health systems are less developed.

## Figures and Tables

**Table 1:** Distribution of answers to the direct question according to the reluctance score (*n* = 160). HCRCT: HIV cure-related clinical trials and ATI: antiretroviral treatment interruptions.

	N	Reluctance Score Mean (SD)	Score > 0 Reluctant	Score ≥ 75th Part of the 25% Most Reluctant	Score ≥ 90th Part of the 10% Most Reluctant	Score ≤ 25th Part of the 25% More Motivated	Score ≤ 10th Part of the 10% More Motivated
If an HCRCT with ATI were available, would you propose it to your patients?							
Probably not	4	0.78 (1.10)	75.00%	50.00%	50.00%	0.00%	0.00%
Yes, perhaps	58	0.43 (1.00)	63.79%	43.10%	18.97%	13.79%	5.17%
Yes, definitely	98	−0.34 (0.83)	30.61%	12.24%	3.06%	34.79%	13.27%

**Table 2:** Factors associated with the reluctance score: results from multivariable linear regressions—acceptability, expectations, and preferences for HCRCT among people living with HIV (PLWH) with undetectable viral load and caregivers, or ANRS-APSEC, study, *n* = 164.

Variables	Initial Multivariable Model (*n* = 154)				Final Model (*n* = 160)		
	β	95% CI	*p*	*p-glob*	β ^a^	95% CI	*p*
**Identity and sociodemographic characteristics**							
Self-identifying as an HIV activist (ref. No)				0.61			
Yes	−0.08	[−0.41; 0.24]	0.61				
**Professional characteristics**							
Number of years since graduating [1; 45 years]	−0.01	[−0.03; 0.01]	0.29	0.29			
Professional activity orientation [0 = care; 10 = research]	−0.05	[−0.13; 0.02]	0.18	0.18	−0.09	[−0.16; −0.02]	0.01
Participated in prevention/information actions organized by HIV associations (ref. No)				0.38			
Yes	−0.14	[−0.47; 0.18]	0.38		−0.33	[−0.61; −0.05]	0.02
Requested to collaborate in writing National HIV guidelines (ref. No)				0.71			
Yes	−0.08	[−0.51; 0.35]	0.71				
**Sources of information about HIV cure research**							
International scientific literature (ref. No)				0.03			
Yes	−0.48	[−0.92; −0.04]	0.03		−0.51	[−0.91; −0.10]	0.01
HIV associations’ journals (ref. No)				0.13			
Yes	−0.26	[−0.59; 0.07]	0.13		−0.33	[−0.61; −0.05]	0.02
Number of sources of information about HIV cure research	−0.07	[−0.20; 0.06]	0.29	0.29			
**Experience with clinical trials**							
Number of clinical trials physician participated in [0; 99]	0.00	[−0.01; 0.01]	0.74	0.74			
Agreement with statement that participation in clinical trials helps advance the work of previous generations (ref. Totally agree)				0.21			
Somewhat agree	0.24	[−0.10; 0.58]	0.17				
Somewhat disagree	−0.25	[−0.83; 0.34]	0.40				
In general, favorable about participation in clinical trials				0.11			
Yes	−0.35	[−0.78; 0.09]	0.11		−0.50	[−0.89; −0.10]	0.01
(Most recent clinical trial) Participated because department head asked them (ref. No)				0.04			
Yes	0.35	[0.02; 0.67]	0.04		0.34	[0.04; 0.64]	0.03
(Most recent clinical trial) Considered having adequately informed patients about benefits and risks (ref. Definitely for both benefits and risks)				0.37			
Somewhat for benefits and/or risks	0.21	[−0.10; 0.52]	0.18				
Not enough for risks and/or benefits	0.26	[−0.36; 0.89]	0.40				
Believed that a cure treatment will become available during their career span (ref. No)				0.03			
Yes	−0.44	[−0.79; −0.09]	0.01				
Do not know	−0.02	[−0.41; 0.36]	0.90				
**Physicians perceptions of their patients’ level of concern about HIV-related difficulties**							
Risk of HIV transmission (ref. Not at all concerned)				0.04			
A little concerned	0.31	[−0.25; 0.86]	0.28				
Very concerned	−0.11	[−0.67; 0.46]	0.72				
Having to live with a secret (ref. Not at all or little concerned)				0.06			
Very concerned	−0.41	[−0.84; 0.02]	0.06		−0.44	[−0.84; −0.04]	0.03
Negative impact on professional life (ref. Not at all concerned)				0.02			
A little concerned	0.73	[0.20; 1.26]	0.01		0.97	[0.49; 1.45]	<01
Very concerned	0.45	[0.03; 0.87]	0.03		0.50	[0.11; 0.89]	0.01
**Confidence in current cART**							
Very confident in the current cART (ref. No)				0.61			
Yes	−0.08	[−0.38; 0.22]	0.61				
Thought that current cART will continue to be effective over the long term (ref. No)				0.55			
Yes	0.14	[−0.33; 0.61]	0.55				

^a^: A positive β is associated with reluctance, and a negative β is associated with agreement. cART: combined antiretroviral therapy.

## Supplementary Materials

The following are available online at https://www.mdpi.com/2076-393X/8/2/334/s1: Figure S1. Description of the three steps of the ANRS-APSEC study. Table S1. Description of the physicians’ characteristics (ANRS-APSEC study, *n* = 164). Table S2. Factors associated with the reluctance score: results from univariable linear regressions (ANRS-APSEC study, *n* = 164).

## Appendix A. Summarized Viewpoints

Respondents were asked:

“On a scale from 1 to 7, how much each of the four following viewpoints shared by some physicians about HIV cure-related clinical trials reflects your own opinion?”

Most motivated*:

“I have confidence in this type of trial and would suggest it even if it brought adverse effects (unless they are too severe) and ART interruption lasted for less than six months. Clear information about the treatment and regular feedback from doctors and even patients will be essential during the trial. My main motivations are to participate in research in order that one day we will be able to avoid the long-term consequences of ART.”

Does not reflect my opinion at all Totally reflects my opinion
1 2 3 4 5 6 7

Moderately motivated*:

“As long as patients can cope well with their current ART, for me to propose a “cure”-type trial, there cannot be any adverse effects. Additionally, it seems to me that these trials are more suitable for people who find it difficult to take their treatment on a daily basis. It will be important to carefully select the target population, to have clear information about the treatments, and that the HIV referral doctor takes care of the trial follow-up himself.”

Does not reflect my opinion at all Totally reflects my opinion
1 2 3 4 5 6 7

Reluctant and benefits-centered*:

“Although it is important to participate in research, and this type of trial could avoid the long-term consequences of ART, suggesting a trial without direct benefits for patients does not make sense. Furthermore, it seems better to me to invest in access to ART for everyone. I would not suggest this type of trial if it caused irreversible adverse effects or if ART interruption did not exceed six months.”

Does not reflect my opinion at all Totally reflects my opinion
1 2 3 4 5 6 7

Reluctant and way of life*:

“Although it is important to make progress for future generations, I do not see the value of proposing a “cure”-type trial that could inhibit the lifestyles of patients who, currently, live almost normally with their ART.”

Does not reflect my opinion at all Totally reflects my opinion
1 2 3 4 5 6 7

* Viewpoint titles were not presented in the questionnaire, only the four descriptions.

## Appendix B. Details Regarding the Construction of the Reluctance Score Using Principal Component Analysis (PCA)

When considering the four viewpoints regarding HCRCT, although the “most motivated” one was the most endorsed by participants with a median Likert scale score of 5 (out of 7) (IQR 4–6), 25% endorsed both the “moderately motivated” and “reluctant and benefits-centered” viewpoints by scoring them from 5 to 7 (out of 7); 25% provided a minimum score of 3 out of 7 for the “reluctant and way of life” viewpoint (Figure A1).

We performed a PCA with a varimax rotation of the physicians’ answers regarding their level of agreement with each of the four viewpoints related to HCRCT (Figure A1). A varimax rotation allows the sum of the variances of the squared loadings to be maximized on each axis.

From the responses, PCA identified only one main factor (i.e., one eigenvalue greater than the value one, according to the Kaiser rule) that explained 52% of the variability with a satisfactory internal consistency with a 0.7 Chronbach alpha (Table A1). Internal consistency was considered satisfactory at values of 0.7 < alpha < 0.8 and acceptable at values of 0.6 < alpha < 0.7 [51,52].

The coordinates of each viewpoint on the axis showed a clear gradient: from a negative coordinate for the most motivated viewpoint to a positive and increasing coordinate for the moderately motivated, reluctant and benefits-centered, and reluctant and way of life viewpoints, resulting in an overall reluctance score. The higher the score, the more reluctant respondents were to propose HCRCT to their patients. The reluctance score regarding HCRCT participation resulting from the PCA of the four viewpoints varied from −1.7 (very motivated) to +3.3 (very reluctant), with a median of −0.15 (IQR −0.69; 0.63).

**Table A1:** Reluctance to propose the HCRCT score: internal consistency and unidimensionality of the items (ANRS-APSEC study, *n* = 164).

Viewpoint	Cronbach Alpha	First Axis
Reluctance score	0.70	52%
Most motivated	0.66	−0.68
Moderately motivated	0.65	0.69
Reluctant & Benefits-centered	0.62	0.75
Reluctant & Way of life	0.59	0.77
