# Supplementary material for: Characterization of Physicians That Might Be Reluctant to Propose HIV Cure-Related Clinical Trials with Treatment Interruption to Their Patients? The ANRS-APSEC Study
Source: Vaccines (Basel). 2020 Jun 23;8(2):334. doi: 10.3390/vaccines8020334 (PMC7350235; doi:10.3390/vaccines8020334)
Supplement: Supplementary file 1 [file vaccines-08-00334-s001.pdf]

Article

Characterization of Physicians That Might Be Reluctant to Propose HIV Cure-Related Clinical Trials with Treatment Interruption to Their Patients? The ANRS-APSEC Study

Christel Protiere [1,*] Lisa Fressard [2], Marion Mora [3], Laurence Meyer [4], Marie Préau [5], Marie Suzan-Monti [6], Jean-Daniel Lelièvre [7], Olivier Lambotte [8], Bruno Spire [9] and the APSEC study group

Supplementary files

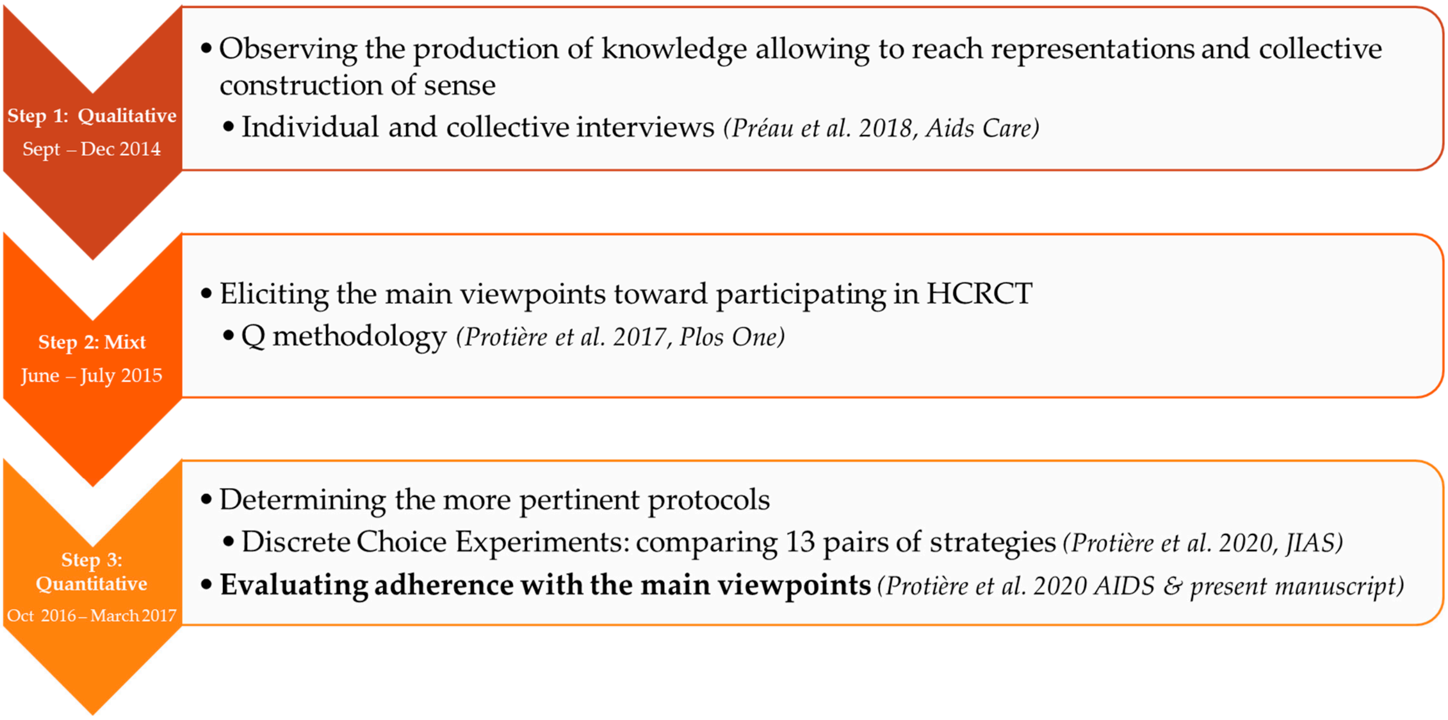

Figure S1: Description of the three steps of the ANRS-APSEC study.

Table S1. Description of physicians' characteristics (ANRS-APSEC study, n=164).

	N	%*
**Identity and sociodemographic characteristics**		
Gender		
Man	80	48.78
Woman	84	51.22
Age (29; 74 years)	164	50 (41; 57)
In a relationship		
Yes	131	79.88
No	33	20.12
Felt part of the LGBT community		
Yes	15	9.15

	N	%*
No	149	90.85
Felt part of the heterosexual community		
Yes	130	79.27
No	34	20.73
Self-identifying as an HIV activist		
Definitely	76	46.34
Somewhat	71	43.29
No	17	10.37
**Professional characteristics**		
Specialty		
HIV specialist (infectiologist, immunologist)	84	51.22
General practitioner	60	36.59
Other (internist, etc.)	20	12.20
Number of years since graduating (1; 45 years)	164	21 (10; 28)
Academic or research involvement		
Yes	64	39.02
No	100	60.98
Orientation of professional activity (0=care; 10=research)	164	3 (2; 5)
Participated in prevention/information actions organized by HIV associations		
Yes	67	40.85
No	97	59.15
Requested to collaborate in writing National HIV guidelines		
Yes	26	15.85
No	138	84.15
**Sources of information about HIV cure research**		
International conferences		
Yes	108	65.85
No	56	34.15
National conferences		
Yes	139	84.76
No	25	15.24
Meetings of learned societies		
Yes	113	68.90
No	51	31.10
International scientific literature		
Yes	143	87.20
No	21	12.80
HIV associations' journals		
Yes	71	43.29
No	93	56.71
Internet websites (ref. No)		
Yes	97	59.15
No	67	40.85
Participation in multidisciplinary networks		
Yes	110	67.07
No	54	32.93
Number of sources of information used about HIV cure research (0; 7)	164	5 (4; 6)
**Experience with clinical trials**		
Number of clinical trials physician participated in (0; 99)	164	20 (10; 40)
Participated in clinical trials to help advance the work of previous generations		
Totally agree	106	64.63
Somewhat agree	46	28.05
Somewhat disagree	12	7.32

	N	%*
General opinion about participating in a clinical trial		
Rather reluctant	2	1.22
Depends of the characteristics of the clinical trial	105	64.02
Deferred to the department head's decision	34	20.73
Favorable	23	14.02
(Most recent clinical trial) Participated in order to advance research		
Yes	129	78.66
No	35	21.34
(Most recent clinical trial) Participated because department head asked them		
Yes	45	27.44
No	119	72.56
(Most recent clinical trial) Participated out of interest		
Yes	110	67.07
No	54	32.93
(Most recent clinical trial) Considered to have adequately informed patients of benefits AND risks—6 mv		
Definitely, for both benefits and risks	58	36.71
Somewhat, for benefits and/or risks	90	56.96
Not enough for benefits and/or risks	10	6.33
**Viewpoints about HIV cure trials**		
Believed that an HIV cure would become available during their career span—6 mv		
Yes	57	34.76
No	67	40.85
Do not know	40	24.39
**Physicians perceptions of their patients' level of concern about HIV-related difficulties**		
Shorter expected life—4 mv		
Very (concerned)	31	19.38
A little (concerned)	87	54.38
Not at all (concerned)	42	26.25
Side-effects related to current cART—4 mv		
Very	77	48.13
Not at all or a little	83	51.88
Severe fatigue—4 mv		
Very	23	14.38
A little	97	60.63
Not at all	40	25.00
Negative impact on health in general—4 mv		
Very	63	39.38
A little	79	49.38
Not at all	18	11.25
Uncertain future—4 mv		
Very	63	39.38
A little	75	46.88
Not at all	22	13.75
Risk of HIV transmission—4 mv		
Very	88	55.00
A little	57	35.63
Not at all	15	9.38
Discrimination—4 mv		
Very	136	85.00
Not at all or a little	24	15.00

	N	%*
Difficulties constructing a stable relationship with a main partner—4 mv		
Very	133	83.13
Not at all or a little	27	16.88
Having to live with a secret—4 mv		
Very	138	86.25
Not at all or a little	22	13.75
Feeling unable to lead a normal life—4 mv		
Very	71	44.38
A little	77	48.13
Not at all	12	7.50
Negative impact on sexuality—4 mv		
Very	129	80.63
Not at all or A little	31	19.38
Negative impact on professional life—4 mv		
Very	29	18.13
A little	106	66.25
Not at all	25	15.63
Cost for society—4 mv		
Very	42	26.25
A little	74	46.25
Not at all	44	27.50
Out-of-pocket expenses—4 mv		
Very	25	15.63
A little	81	50.63
Not at all	54	33.75
**Physicians perceptions of patients' lives with cART**		
Believed that having to take medication on a daily basis is easy for patients—4 mv		
Yes	77	48.13
No	83	51.88
Opinion of ART-related side effects for patients—4 mv		
Not at all uncomfortable	12	7.50
A little uncomfortable	126	78.75
Very uncomfortable	22	13.75
**Confidence in current cART**		
Very confident in current cART—4 mv		
Yes	87	53.05
No	77	46.95
Believed that current ART would continue to be effective over the long term—4 mv		
Yes	142	86.59
No	22	13.41

* or median (IQR).
mv: missing values.

Table S2. Factors associated with the reluctance score: results from univariable linear regressions (ANRS-APSEC study, n=164).

	β	95% CI	*p*	*p-glob*
Identity and sociodemographic characteristics				

	β	95% CI	*p*	*p-glob*
Gender (ref. Man)				0.87
Woman	0.03	[-0.28;0.34]	0.87	
Age [29;74 years]	-0.01	[-0.02;0.01]	0.45	0.45
In a relationship (ref. No)				0.86
Yes	0.03	[-0.35;0.42]	0.86	
Felt part of the LGBT community (ref. No)				0.45
Yes	0.21	[-0.33;0.74]	0.45	
Felt part of the heterosexual community (ref. No)				0.67
Yes	-0.08	[-0.46;0.30]	0.67	
Self-identifying as an HIV activist (ref. Definitely)				
Somewhat	0.26	[-0.06;0.59]	0.11	0.28
No	0.13	[-0.40;0.66]	0.62	
Definitely identifying themselves as an HIV activist (ref. No)				0.13
Yes	-0.24	[-0.55;0.07]	0.13	
Professional characteristics				
Specialty (ref. HIV specialist (infectiologist, immunologist))				
General practitioner	-0.17	[-0.51;0.16]	0.30	0.43
Other (internist, etc.)	-0.26	[-0.75;0.23]	0.30	
Number of years since graduating [1;45 years]	-0.01	[-0.02;0.00]	0.14	0.14
Academic or research involvement (ref. No)				0.45
Yes	-0.12	[-0.44;0.20]	0.45	
Orientation of professional activity [0=care;10=research]	-0.12	[-0.19;-0.05]	0.001	0.001
Participated in prevention/information actions organized by HIV associations (ref. No)				0.01
Yes	-0.43	[-0.74;-0.12]	0.01	
Requested to collaborate in writing National HIV guidelines (ref. No)				0.10
Yes	-0.35	[-0.77;0.07]	0.10	
Sources of information used about HIV cure research				
International conferences (ref. No)				0.40
Yes	-0.14	[-0.47;0.19]	0.40	
National conferences (ref. No)				0.27
Yes	-0.24	[-0.67;0.19]	0.27	
Meetings of learned societies (ref. No)				0.22
Yes	-0.21	[-0.54;0.12]	0.22	
International scientific literature (ref. No)				0.04
Yes	-0.48	[-0.94;-0.02]	0.04	
HIV associations' journals (ref. No)				0.06
Yes	-0.30	[-0.61;0.01]	0.06	
Internet websites (ref. No)				0.38
Yes	-0.14	[-0.45;0.17]	0.38	
Participation in multidisciplinary networks (ref. No)				0.43
Yes	-0.13	[-0.46;0.20]	0.43	
Number of sources of information about HIV cure research	-0.15	[-0.26;-0.04]	0.01	0.01
Experience with clinical trials				
Number of clinical trials physician participated in [0;99]	-0.01	[-0.02;0.00]	0.01	0.01
Agreement with statement that participation in clinical trials helps to advance the work of previous generations (ref. Totally agree)				0.08
Somewhat agree	0.24	[-0.11;0.58]	0.18	
Somewhat disagree	-0.47	[-1.07;0.12]	0.12	

	β	95% CI	*p*	*p-glob*
General attitude about participating in a clinical trial				
Favorable (ref. No)				0.004
Yes	-0.65	[-1.08;-0.21]	0.004	
Rather reluctant (ref. No)				0.62
Yes	0.36	[-1.05;1.77]	0.62	
Depends on characteristics of the clinical trial				0.40
Yes	0.14	[-0.18;0.46]	0.40	
Deferred to the department head's decision				0.19
Yes	0.25	[-0.12;0.63]	0.19	
Reason for participation in the most recent clinical trial				
In order to advance research (ref. No)				0.202
Yes	-0.24	[-0.62;0.13]	0.20	
Because department head asked them (ref. No)				0.18
Yes	0.23	[-0.11;0.58]	0.18	
Out of interest (ref. No)				0.79
Yes	-0.04	[-0.37;0.28]	0.79	
(Most recent clinical trial) Considered to have adequately informed patients of both the benefits and risks of the trial (ref. Definitely, for both benefits and risks)—6 mv				0.04
Somewhat, for benefits or/and risks	0.37	[0.04;0.70]	0.03	
Not enough for risks or/and benefits	0.63	[-0.04;1.30]	0.07	
Believed an HIV cure would become available during their career span (ref. No)				0.06
Yes	-0.25	[-0.61;0.10]	0.15	
Do not know	0.23	[-0.16;0.62]	0.25	
Physicians' perceptions of their patients' level of concern about HIV-related difficulties				
Shorter expected life (ref. Not at all)—4 mv				
A little (concerned)	0.19	[-0.17;0.56]	0.29	0.57
Very (concerned)	0.11	[-0.35;0.56]	0.64	
Side effects related to actual ART (ref. Not at all or a little)—4 mv				0.66
Very	-0.07	[-0.37;0.24]	0.66	
Severe fatigue (ref. Not at all)—4 mv				
A little	-0.23	[-0.59;0.13]	0.22	0.29
Very	0.06	[-0.45;0.56]	0.83	
Negative impact on health in general (ref. Not at all)—4 mv				
A little	0.28	[-0.22;0.79]	0.27	0.50
Very	0.29	[-0.22;0.81]	0.26	
Uncertain future (ref. Not at all)—4 mv				
A little	0.29	[-0.17;0.76]	0.22	0.35
Very	0.35	[-0.13;0.82]	0.15	
Risk of HIV transmission (ref. Not at all)—4 mv				
A little	0.16	[-0.39;0.71]	0.58	0.04
Very	0.51	[-0.02;1.04]	0.06	
Discrimination (ref. Not at all or a little—4 mv				0.65
Very	-0.10	[-0.53;0.33]	0.65	
Difficulties constructing a stable relationship with a main partner (ref. Not at all or a little)—4 mv				0.38
Very	-0.18	[-0.59;0.22]	0.38	
Having to live with a secret (ref. Not at all or a little)—4 mv				0.13
Very	-0.34	[-0.78;0.10]	0.13	

	β	95% CI	*p*	*p-glob*
Feeling unable to lead a normal life (ref. Not at all)—4 mv				
A little	0.17	[-0.43;0.77]	0.57	0.84
Very	0.17	[-0.43;0.77]	0.58	
Negative impact on sexuality (ref. Not at all or a little)—4 mv				0.51
Very	0.13	[-0.26;0.51]	0.51	
Negative impact on professional life (ref. Not at all)—4 mv				
A little	0.30	[-0.12;0.73]	0.16	0.06
Very	0.63	[0.11;1.15]	0.02	
Cost for society (ref. Not at all)—4 mv				
A little	-0.08	[-0.45;0.29]	0.68	0.91
Very	-0.07	[-0.49;0.34]	0.73	
Out-of-pocket expenses (ref. Not at all)—4 mv				
A little	-0.14	[-0.48;0.20]	0.42	0.72
Very	-0.06	[-0.52;0.41]	0.81	
Perception of patients' life with cART				
Believed that having to take medication on a daily basis is easy for patients (ref. No)—4 mv				0.74
Yes	0.05	[-0.25;0.36]	0.74	
Opinion on ART-related side effects for patients (ref. Not at all uncomfortable)—4 mv				0.71
A little uncomfortable	0.00	[-0.58;0.58]	1.00	
Very uncomfortable	-0.19	[-0.88;0.51]	0.60	
Confidence in current cART				
Very confident in current cART (ref. No)				0.05
Yes	-0.31	[-0.62;-0.01]	0.05	
Believed that current cART will continue to be effective over the long term (ref. No)				0.11
Yes	-0.37	[-0.82;0.08]	0.11	

mv: missing
